# New Bills of Mortality for London

**Published:** 1842-01

**Authors:** 


### PART FOURTH.

### Medical Intelligence.

### TABLE OF MORTALITY FOR LONDON,

### For the Third Quarter of 1841:

Showing the number of Deaths from all causes registered in thirteen weeks, from the 24th June, 1841, to the 25th September, 1841.

| Causes of Death. | Total Deaths. |
|---|---|
| **Class I.** | |
| Smallpox | 142 |
| Measles | 249 |
| Scarlatina | 199 |
| Hooping-cough | 452 |
| Croup | 74 |
| Thrush | 94 |
| Diarrhœa | 200 |
| Dysentery | 28 |
| Cholera | 22 |
| Influenza | 8 |
| Typhus | 302 |
| Erysipelas | 46 |
| Syphilis | 11 |
| Hydrophobia | 0 |
| Total Epidemic, &c. | 1814 |
| **Class II.** | |
| Cephalitis | 152 |
| Hydrocephalus | 443 |
| Apoplexy | 186 |
| Paralysis | 140 |
| Convulsions | 780 |
| Epilepsy | 38 |
| Insanity | 7 |
| Delirium tremens | 22 |
| Dis. of brain, &c. | 114 |
| Total Dis. of Brain, &c. | 1882 |
| **Class III.** | |
| Quinsy | 15 |
| Bronchitis | 87 |
| Pleurisy | 16 |
| Pneumonia | 721 |
| Hydrothorax | 37 |
| Asthma | 115 |
| Consumption | 1828 |
| Dis. of lungs, &c. | 131 |
| Total Dis. of Lungs, &c. | 2950 |

| Causes of Death. | Total Deaths. |
|---|---|
| **Class IV.** | |
| Pericarditis | 5 |
| Aneurism | 7 |
| Dis. of the heart, &c. | 208 |
| Total Dis. of Heart, &c. | 220 |
| **Class V.** | |
| Teething | 333 |
| Gastritis, enteritis | 310 |
| Peritonitis | 9 |
| Tabes mesenterica | 72 |
| Ascites | 9 |
| Ulceration | 16 |
| Hernia | 23 |
| Colic or ileus | 30 |
| Dis. of stomach, &c. | 74 |
| Hepatitis | 19 |
| Jaundice | 21 |
| Dis. of the liver, &c. | 122 |
| Total Dis. of Stom. &c. | 938 |
| **Class VI.** | |
| Nephritis | 5 |
| Diabetes | 2 |
| Stone | 6 |
| Stricture | 5 |
| Dis. of the kidneys, &c. | 34 |
| Tot. Dis. of Kidneys, &c. | 52 |
| **Class VII.** | |
| Childbed | 61 |
| Ovarian dropsy | 8 |
| Diseases of uterus, &c. | 37 |
| Tot. Dis. of Uterus, &c. | 106 |
| **Class VIII.** | |
| Rheumatism | 31 |
| Diseases of joints, &c. | 27 |
| Total Dis. of Joints, &c. | 58 |

| Causes of Death. | Total Deaths. |
|---|---|
| **Class IX.** | |
| Ulcer | 4 |
| Fistula | 3 |
| Diseases of skin, &c. | 5 |
| Total Dis. of Skin, &c. | 12 |
| **Class X.** | |
| Inflammation | 54 |
| Hemorrhage | 35 |
| Dropsy | 365 |
| Abscess | 47 |
| Mortification | 52 |
| Scrofula | 30 |
| Carcinoma | 97 |
| Tumour | 25 |
| Gout | 10 |
| Atrophy | 126 |
| Debility | 262 |
| Malformations | 7 |
| Sudden deaths | 146 |
| T. Dis. of Uncert. Seat | 1256 |
| **Class XI.** | |
| Old age or nat. decay | 653 |
| **Class XII.** | |
| Intemperance | 11 |
| Privation | 6 |
| Violent deaths | 259 |
| Total by Violence, &c. | 276 |
| **Class XIII.** | |
| Causes not specified | 53 |
| T. Deaths from all causes, Males 5343, Females 4931 | 10274 |

### TEMPERATURE DURING THE QUARTER.

| | Weeks ending | | | | | | | | | | | | |
|---|---|---|---|---|---|---|---|---|---|---|---|---|---|
| | July | | | | | August | | | | September | | | |
| | 3d | 10th | 17th | 24th | 31st | 7th | 14th | 21st | 28th | 4th | 11th | 18th | 25th |
| Highest | 74 | 80 | 75 | 74 | 71 | 71 | 73 | 76 | 77 | 77 | 60 | 70 | 70 |
| Lowest | 53 | 52 | 50 | 51 | 52 | 50 | 51 | 53 | 51 | 51 | 43 | 50 | 51 |
| Daily mean | 62 | 65 | 60 | 60 | 60 | 63 | 63 | 64 | 63 | 64 | 57 | 66 | 61 |

Quarterly Maximum .... 80° Minimum .... 43° Daily Mean .... 62°

